# Genomic Insights of *Cryobacterium* Isolated From Ice Core Reveal Genome Dynamics for Adaptation in Glacier

**DOI:** 10.3389/fmicb.2020.01530

**Published:** 2020-07-14

**Authors:** Yongqin Liu, Liang Shen, Yonghui Zeng, Tingting Xing, Baiqing Xu, Ninglian Wang

**Affiliations:** ^1^Key Laboratory of Tibetan Environment Changes and Land Surface Processes, Institute of Tibetan Plateau Research, Chinese Academy of Sciences, Beijing, China; ^2^CAS Center for Excellence in Tibetan Plateau Earth Sciences, Beijing, China; ^3^University of Chinese Academy of Sciences, Beijing, China; ^4^College of Life Sciences, Anhui Normal University, Wuhu, China; ^5^Department of Environmental Science, Aarhus University, Roskilde, Denmark; ^6^College of Urban and Environmental Science, Northwest University, Xi’an, China

**Keywords:** glacier, *Cryobacterium*, genomic, evolutionary processes, cold adaptation

## Abstract

Glacier is the dominant cold habitat in terrestrial environments, providing a model ecosystem to explore extremophilic strategies and study early lives on Earth. The dominant form of life in glaciers is bacteria. However, little is known about past evolutionary processes that bacteria underwent during adaptation to the cryosphere and the connection of their genomic traits to environmental stressors. Aiming to test the hypothesis that bacterial genomic content and dynamics are driven by glacial environmental stressors, we compared genomes of 21 psychrophilic *Cryobacterium* strains, including 14 that we isolated from three Tibetan ice cores, to their mesophilic counterparts from the same family Microbacteriaceae of Actinobacteria. The results show that psychrophilic *Cryobacterium* underwent more dynamic changes in genome content, and their genomes have a significantly higher number of genes involved in stress response, motility, and chemotaxis than their mesophilic counterparts (*P* < 0.05). The phylogenetic birth-and-death model imposed on the phylogenomic tree indicates a vast surge in recent common ancestor of psychrophilic *Cryobacterium* (gained the greatest number of genes by 1,168) after the division of the mesophilic strain *Cryobacterium mesophilum*. The expansion in genome content brought in key genes primarily of the categories “cofactors, vitamins, prosthetic groups, pigments,” “monosaccharides metabolism,” and “membrane transport.” The amino acid substitution rates of psychrophilic *Cryobacterium* strains are two orders of magnitude lower than those in mesophilic strains. However, no significantly higher number of cold shock genes was found in psychrophilic *Cryobacterium* strains, indicating that multi-copy is not a key factor for cold adaptation in the family Microbacteriaceae, although cold shock genes are indispensable for psychrophiles. Extensive gene acquisition and low amino acid substitution rate might be the strategies of psychrophilic *Cryobacterium* to resist low temperature, oligotrophy, and high UV radiation on glaciers. The exploration of genome evolution and survival strategies of psychrophilic *Cryobacterium* deepens our understanding of bacterial cold adaptation.

## Introduction

Glaciers and ice sheets have been recognized as biomes, uniquely dominated by microorganisms (Hodson et al., 2008; Anesio and Laybourn-Parry, 2012; Grinsted, 2013). Microbial metabolism occurs on glaciers and many community members not only perform basal metabolic functions but also grow and divide (Price, 2000). Microorganisms living in these environments have evolved unique features in their proteins, enzyme, membranes, and genetic responses to low temperature and nutrient concentrations and excessive UV radiation (Siddiqui et al., 2013; De Maayer et al., 2014).

Genomic survival strategies have been investigated in a few glacial model organisms. Genes encoding proteins with known or predicted roles in cold adaptation, i.e., cold-shock protein, proteorhodopsin, osmoprotection, and membrane-related proteins, were found in genomes of psychrophilic species *Flavobacterium bomense* sp. nov. isolated from glaciers (Liu et al., 2019a). Genome of an ice core strain *Dyadobacter tibetensis* Y620-1 contained high percentage of new novel genes and genes required for the serine-glyoxylate cycle in one-carbon metabolism, which may contribute to its survival in glacier (Shen et al., 2019). Although glacier bacteria and psychrophiles from arctic soil are both cold acclimated, glacier bacteria show different genomic adaptation characteristics mainly connected to the genes devoted to CRISPR (Clustered Regularly Interspaced Short Palindromic Repeats) defense system, osmotic adaptation, and metabolism of monosaccharides, nitrogen, and aromatic compounds, due to the different environmental pressures experienced by glacier bacteria and psychrophiles from arctic soils (Shen et al., 2017). The survival strategies of psychrophiles at the genome level have been well investigated during past decades (Saunders et al., 2003; Methé et al., 2005; De Maayer et al., 2014; Raymond-Bouchard et al., 2018). However, the role of microevolution, genomic adaptive strategies, and environmental factors in shaping the genomes of bacteria colonizing glaciers are largely unknown. Glaciers not only are the dominant cold habitat to explore the extremophilic strategies in the terrestrial land but also are the representative habitats of early lives on the Earth and perhaps on other planets as well (Priscu et al., 2004).

Species of *Cryobacterium* are widely distributed in cold environments and well adapted to cold conditions (Liu et al., 2018). The genus *Cryobacterium*, proposed by Suzuki et al. (1997), is consisting of Gram-positive aerobes that have a pleomorphic rod-shaped morphology. The type species of *Cryobacterium psychrophilum* is an obligate psychrophilic actinomycete. At the time of writing, the genus *Cryobacterium* comprises 11 recognized species, *C. psychrophilum* (Suzuki et al., 1997; Inoue and Komagata, 2006), *Cryobacterium psychrotolerans* (Zhang et al., 2007), *Cryobacterium mesophilum* (Dastager et al., 2008), *Cryobacterium roopkundense* (Reddy et al., 2010), *Cryobacterium arcticum* (Bajerski et al., 2011), *Cryobacterium flavum* and *Cryobacterium luteum* (Liu et al., 2012), *Cryobacterium levicorallinum* (Liu et al., 2013), *Cryobacterium zongtaii* (Liu et al., 2018), *Cryobacterium soli* (Gong et al., 2019), and *Cryobacterium melibiosiphilum* (Liu et al., 2019b). All type strains of *Cryobacterium* except *C. mesophilum* described by Dastager et al. (2008) were isolated from cold environments and recognized as psychrophiles with optional growth temperature ranging from 15 to 20°C and growth occurs between 0 and 25°C. The strain *C. mesophilum* grew between 20 and 28°C, with optimum growth occurring at 25–28°C (Dastager et al., 2008). Phenotypic analysis showed that the genus *Cryobacterium* differed from its Microbacteriaceae counterparts in the presence of a significant amount of 12-methyl pentadecenoic acid (i.e., a-15:l). The presence of a-15:l is unusual but reasonable for psychrophilic Gram-positive bacteria in order to maintain membrane fluidity at low temperatures (Suzuki et al., 1997).

Most of the reported novel *Cryobacterium* species were isolated from glacial environments (Liu et al., 2018). The culturable bacteria in two ice cores from the Tibetan Plateau were dominated by *Cryobacterium* (Liu et al., 2019c). *Cryobacterium* exhibited high diversity in more than 10 glaciers around world (Segawa et al., 2005; Liu et al., 2016, 2018), indicating that *Cryobacterium* species have developed strategies to endure the harsh glacier habitats. Previous studies were most focused on adaptation features of *Cryobacterium* isolates to cold environments using polyphasic and multilocus sequence analysis (Sathyanarayana Reddy et al., 2014; Singh et al., 2015; Lee et al., 2016; Liu et al., 2018). However, a detailed comparative genomic study of multiple *Cryobacterium* genomes is lacking, which could contribute markedly to and validate our understanding of molecular strategies underlying this genus’ cold adaptation. In the present study, we analyzed the genomes of 21 psychrophilic *Cryobacterium* isolates of ice core origin in comparison to their mesophilic counterparts with the aim to deepen our understanding of how bacteria adapt to glacial environments. Our data suggest that the combination of comparative genomics approach and biogeography can be a powerful tool to decipher the bacterial cold adaptation mechanisms in psychrophiles.

## Materials and Methods

Fourteen psychrophilic *Cryobacterium* strains were selected from our culture collection including five strains isolated from the Muztag Ata glacier (named M series, mean annual air temperature −4°C, 36.4° N, 87.3° E, 6,973 m a.s.l.), three isolates from the Noijin Kangsang glacier (N, mean annual air temperature −8°C, 90.2° E, 29.0° N, 5,950 m a.s.l.), and six isolates from the Yuzhufeng glacier (Y, mean annual air temperature −5°C, 35.5° N, 94.2° E, 6,178 m a.s.l.), respectively (Table 1) (Liu et al., 2019c). Growth at various temperatures (0–40°C, with an interval of 5°C) was measured (0°C was maintained with an ice–water mixture and the remaining settings were achieved using a constant-temperature incubator). OD_600_ was measured with a micro plate reader (MD Spectra Max M5) to assess the growth. Growth of these strains occurred between 0 and 25°C with optimum growth at 18–20°C.

**TABLE 1:** Information of the genomes of 21 psychrophilic and one mesophilic *Cryobacterium*.

**Strain ID**	**Size (Mbp)**	*CDSs	**RNAs**	**Location	**G + C% mol**	**CRISPRs**	**Cold Shock**	**Completeness (%)**	**Specific gene families**
M15	3.67	3497	49	Muztag Ata	63.4	0	2	98.99	1070
M23	3.55	3347	52	Muztag Ata	66.8	1	2	99.49	1029
M25	3.35	3177	53	Muztag Ata	67.0	0	2	99.49	949
M91	3.99	3822	48	Muztag Ata	64.2	0	2	98.99	1338
M96	3.30	3096	51	Muztag Ata	67.0	1	2	99.49	858
N19	4.29	4070	50	Noijin Kangsang	64.5	0	2	98.99	1420
N21	4.14	3944	53	Noijin Kangsang	64.4	1	2	98.99	1429
N22	4.06	3751	53	Noijin Kangsang	68.3	0	2	99.37	803
Y11	4.03	3891	51	Yuzhufeng	63.3	3	2	99.24	1114
Y29	3.66	3484	49	Yuzhufeng	63.5	0	2	98.99	1059
Y50	4.40	4344	49	Yuzhufeng	63.2	0	2	98.65	1567
Y57	4.01	3884	50	Yuzhufeng	63.2	0	2	98.99	1196
Y62	4.23	4179	48	Yuzhufeng	63.2	0	2	99.49	1559
Y82	3.69	3556	50	Yuzhufeng	63.6	0	2	98.74	1008
CGMCC 1.11215	4.04	3906	51	No.1 Glacier	64.7	0	2	98.99	928
CGMCC 1.11211	3.75	3531	51	No.1 Glacier	64.5	0	2	98.74	805
CGMCC 1.11210	3.83	3600	52	No.1 Glacier	65.1	0	2	98.99	719
CGMCC 1.5382	3.25	3016	51	No.1 Glacier	68.3	0	2	96.49	350
RuG17	4.36	4048	50	Himalaya	65.3	3	2	98.74	776
PAMC 27867	4.17	3826	61	Antarctic	68.6	1	2	99.49	692
MLB-32	4.27	3214	72	Arctic	64.9	0	2	96.49	804
^§^ CGMCC 1.10440	2.41	2342	48	Korea	66.5	0	2	96.04	143

*CDSs, coding sequences; **all the strains isolated from Tibetan Plateau except where noted; ^§^mesophilic Cryobacterium strain.

For genome sequencing, high-quality genomic DNA was extracted from cells grown on R2A for 3 days at 20°C using TIANamp Bacteria DNA Kit (TIANGEN, Beijing) following the manufacturer’s instructions. The purity of genomic DNA was assessed with NanoDrop (2000c, Thermo Scientific, United States) and all had an OD 260:280 ratio of 1.8–2.0. DNA was stored in TE buffer (pH 8.0) for genome sequencing.

Sequencing was performed on an Illumina Hiseq 2000 instrument. Reads were assembled using SPAdes v3.11.1 with default options (Bankevich et al., 2012). As the algorithm is sensitive to sequencing errors, low-quality reads were filtered prior to *de novo* assembly using Fastp with default options (Chen et al., 2018). The genome sequences were deposited at DDBJ/ENA/GenBank under accession numbers PJJJ00000000-PJJX00000000. The version described in this paper is PJJJ01000000-PJJX01000000.

In addition to the 14 *Cryobacterium* genomes sequenced in this study, 18 genomes were downloaded from the NCBI genome database (last access in April 2017, Supplementary Table S1). These include seven psychrophilic *Cryobacterium* strains isolated from the No. 1 Glacier in China and soils from Himalaya, Arctic, and Antarctic (Liu et al., 2013, 2018); one mesophilic *Cryobacterium* strain from a Korean soil (Dastager et al., 2008); and 10 genomes in the family *Microbacteriaceae* and 2 *Rubrobacter* genomes. At the time of writing, there was only one mesophilic strain’s genome available in the genus *Cryobacterium*, and thus we included 10 type strains in the family Microbacteriaceae that were phylogenetically close to *Cryobacterium* in order to perform a statistically sound comparative analysis. These 34 genomes were divided into two groups: the psychrophilic *Cryobacterium* genomes (referencing to strains from poles and Tibetan Plateau), and the reference genomes (mesophiles, referencing to *C. mesophilum* CGMCC 1.10440^T^ and the 10 genomes in the family *Microbacteriaceae*). Two strains (*Rubrobacter radiotolerans* RSPS-4 and *Rubrobacter xylanophilus* DSM9941) were used as out-group in the phylogenetic analysis; the out-group strains were not included in the comparative genomics analysis.

The completeness of genomes was calculated using CheckM (Parks et al., 2015). 16S rRNA genes for phylogenetic analysis were generated from the genomes using RNAmmer (v.1.2) (Lagesen et al., 2007). To remove potential differences introduced through different annotation methods, all genomes were re-annotated in the present study with RAST (Rapid Annotation using Subsystem Technology) (Overbeek et al., 2014). An all-versus-all search was performed with BLAST + 2.2.28, with an *E*-value cutoff of 1e−5. Genes without orthologs (pv_cutoff 1-e5 –pi_cutoff 70 –pmatch_cutoff 70) were considered as specific genes. CRISPRs were identified with CRISPR-finder (Grissa et al., 2007a). Codon usage, amino acid composition and protein comparisons between genomes were carried out with the PERL scripts “CodonAaUsage.pl,” “aminoacidUsage.pl,” and the programs “matrix_createConfig” and “matrix” (Vesth et al., 2013). Heatmaps were produced with R (Ihaka and Gentleman, 1996). Multiple alignments were performed using ClustalW, and topology trees of the 16S rRNA genes and functional genes were constructed using MEGA v10.0.5 with bootstrap method (1,000 iterations) (Kumar et al., 2018).

The same set of 32 genomes was used for gene family clustering analysis (*R. radiotolerans* RSPS-4 and *R. xylanophilus* DSM9941 served as out-groups). An all-versus-all BLAST search was performed, and the FastOrtho software (–pv_cutoff 1-e5 –pi_cutoff 70 –pmatch_cutoff 70)^1^ was used to identify gene families. The output of FastOrtho analysis was parsed using custom-made PERL scripts. Gene families that were not present in any other genomes were considered as strain-specific gene families. One hundred and nine shared orthologs were identified at the amino acid level using an *E*-value threshold of 10^–5^ and 74 of them were single-copy orthologs. Then, the 74 single-copy orthologs were concatenated using custom-made PERL scripts to perform phylogeny construction. The concatenated sequences and the detailed description of the genes are available in the Supplementary Table S2. These genes include RNA polymerase sigma factor *rpoD*, enolase, translation elongation factor G (EF-G) and DNA gyrase subunit A (*gyrA*), which are core genes of bacteria (Gil et al., 2004).

As the first step for genome tree construction, the concatenated orthologous genes were aligned at the amino acid sequence level using the Muscle software v3.8.31 (Edgar, 2004). Then non-conserved segments in the alignments were trimmed using the Gblock (Castresana, 2000) software with all gap-containing columns discarded using the parameter values (-b1 = 50 -b4 = 5, and other parameters as default values) as suggested by Luo et al. (2013). Secondly, two probabilistic phylogenetic approaches were used to analyze the concatenation data (19,306 sites) of the 74 homologs. One is a maximum-likelihood (ML) method (Felsenstein, 1981) using the MPI version of RAxML v8.2.4 software (Stamatakis, 2014) and the other is Bayesian method using the MPI version of MrBayes v3.2.6 (Ronquist and Huelsenbeck, 2003). As the evolutionary models for different sites in multi-gene concatenated alignments may differ, the PartitionFinder software v2.1.0 (Lanfear et al., 2012) was used to identify the best scheme for RAxML and MrBayes. Amino acid substitution rate was extracted from the ML tree.

The 34 genomes were further used for ancestral reconstruction. The out-group species Rhodothermaceae bacterium and *Pseudoclavibacter soli* DSM 23366 were not included because the reconstruction of ancestral genome content using COUNT does not require out-group species (Csuroes, 2010). Maximum-likelihood birth-and-death models and the Dollo parsimony method implemented in the COUNT software were used to reconstruct the size of ancestral gene families. Ancestral history reconstruction was performed using posterior probabilities: 100 rounds of rate optimization were computed with a convergence threshold of 10^–3^ prior to ancestral reconstruction; other parameters were set as default as suggested by Oliveira et al. (2017). The genomes were split into subcategories, and calculations and text processing, such as extracting sequences from genome files and parsing BLAST outputs, were performed using custom-made PERL scripts, which are available from the authors on request.

## Results

### Overview of *Cryobacterium* Genomes From Tibetan Plateau Glaciers

The genome size of 14 *Cryobacterium* stains from Tibetan Plateau glaciers range from 3.30 Mbp (3,096 protein coding sequences) to 4.40 Mbp (4,344 protein coding sequences) with an average of 3.88 Mbp (3,614 protein coding sequences) (Table 1). The genomic GC content ranged from 63.2 to 68.3%, with an average of 64.7%. The number of strain-specific gene families ranged from 803 to 1,567, and the number of rRNA genes differed from 48 to 53. All 14 strains have 2 predicted cold shock genes and 14 heat shock genes. CRISPRs were identified in four strains, one in each of strains M23, M96, and N21 and three in strain Y11, lower than the average occurrence frequency (∼39%) of CRISPR loci in the sequenced bacterial genomes (Grissa et al., 2007b).

### Phylogeny Reconstruction

**FIGURE 1:**
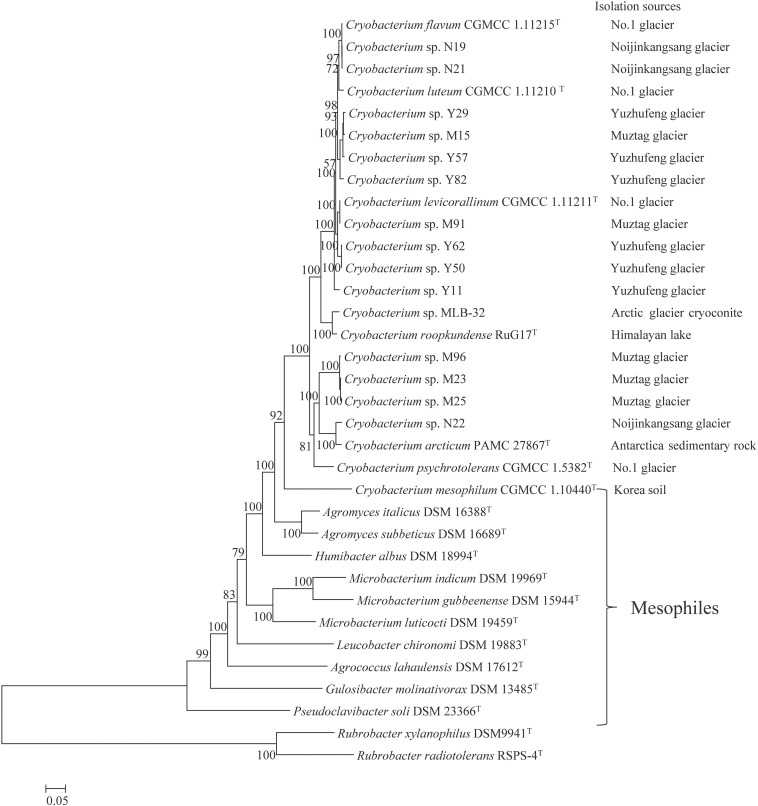
Phylogenetic clustering of 32 Microbacteriaceae strains. Phylogeny is based on concatenated alignment of 74 orthologous proteins with 1,000 bootstraps using RAxML. *Rubrobacter xylanophilus* DSM9941 and *R. radiotolerans* RSPS-4 were used as out-groups. Bar represents accumulated changes per amino acid.

The maximum-likelihood tree based on 74 concatenated single-copy orthologous genes shows that the *Cryobacterium* species are well separated from the mesophilic Microbacteriaceae species (Figure 1 and Supplementary Figure S1). The 14 strains from the Tibetan Plateau glacier are placed in five different branches, clustered into lineage 1 (N19 and N21), lineage 2 (Y29, M15, Y57, and Y82), lineage 3 (M91, Y62, and Y50), lineage 4 (M96, M23, M25, and N22), and strain Y11 forms a branch with a single lineage. Each lineage includes a type strain except one lineage composed by Y29, M15, Y57, and Y82. All lineages have strains isolated from different sources, and the most diverse branch contained strains isolated from four sources, i.e., Muztag Ata (M96, M23, and M25), NJKS (N21), No. 1 Glacier (*C. psychrotolerans* CGMCC 1.5832^T^), and Antarctic (*C. arcticum* PAMC 27867^T^, PAMC represents Polar and Alpine Microbial Collection).

### Features in Main Functional Categories and Amino Acid Composition

**FIGURE 2:**
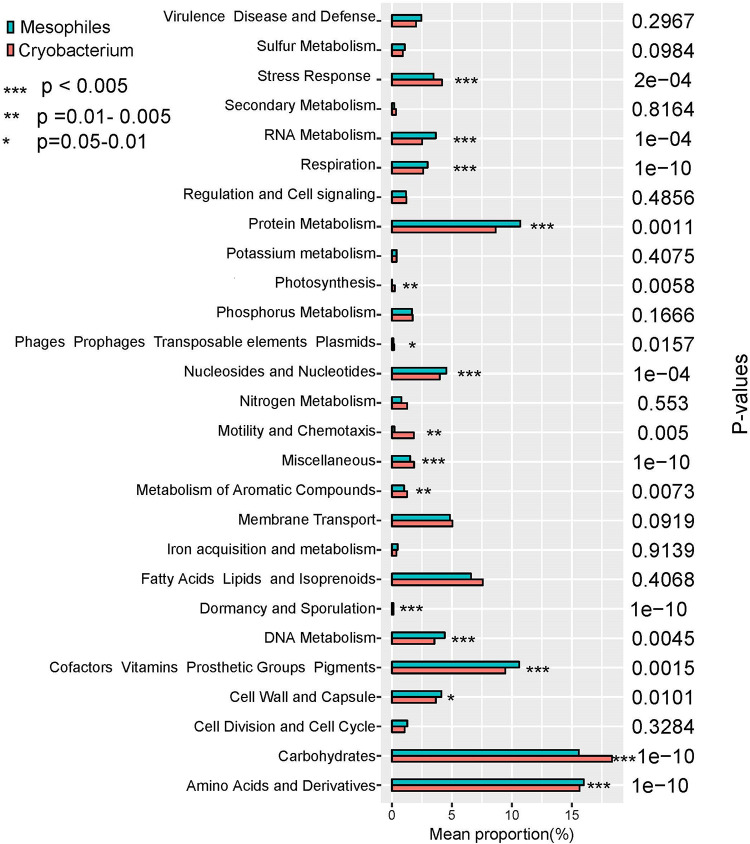
Proportional differences of functional category between psychrophilic *Cryobacterium* strains and those of reference mesophilic strains, *P*-values were obtained using one-way ANOVA. *Cryobacterium* genomes from poles and Tibetan Plateau represent the psychrophilic group, and the mesophiles’ genomes include *C. mesophilum* CGMCC 1.10440^T^ and the 10 genomes in the family Microbacteriaceae as described in Section “Materials and Methods.”

The percentages of gene functional categories were calculated based on RAST annotation system. Genes related to stress response, photosynthesis, motility and chemotaxis, metabolism of aromatic compounds, dormancy and sporulation, and carbohydrates increased significantly in proportion (*P* < 0.05, Figure 2) in *Cryobacterium* compared to reference strains.

Genes involved in “RNA metabolism,” “protein metabolism,” “nucleosides and nucleotides,” “DNA metabolism,” “cofactors, vitamins, prosthetic groups, pigments,” “cell wall and capsule,” and “amino acids and derivatives” decreased significantly (*P* < 0.05, Figure 2) in proportion in *Cryobacterium* compared to the reference strains. Genes involved in “virulence disease and defense,” “sulfur metabolism,” “regulation and cell signaling,” “potassium metabolism,” “phosphorus metabolism,” “membrane transport,” “iron acquisition and metabolism,” “fatty acids lipids and isoprenoids,” and “cell division and cell cycle” did not show significant differences (*P* > 0.05, Figure 2).

**FIGURE 3:**
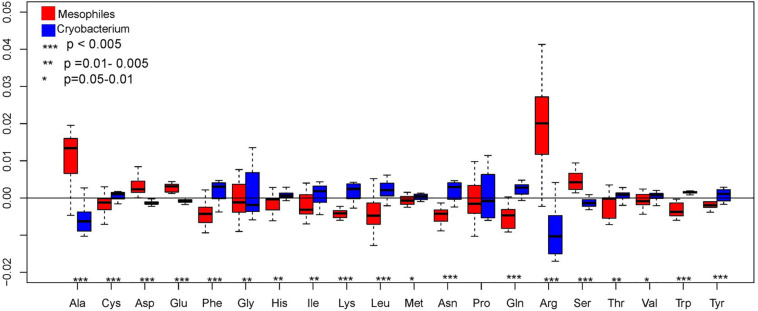
Box plot showing the comparison of genome-wide amino acid composition between *Cryobacterium* strains and reference strains, *P*-values were obtained using one-way ANOVA. *Cryobacterium* genomes from poles and Tibetan Plateau represent the psychrophilic group and the mesophiles’ genomes include *C. mesophilum* CGMCC 1.10440^T^ and the 10 genomes in the family Microbacteriaceae as described in Section “Materials and Methods.”

The pattern of amino acid distribution in *Cryobacterium* spp. displays an overall similar trend in their genomes, with Arg being the most abundant, followed by Ala, Gly, and Pro, while Met, Lys, and Tyr were infrequent. Composition of Ala, Asp, Glu, Arg, and Ser decreased significantly in proportion in psychrophilic *Cryobacterium* strains compared to reference strains (one-way ANOVA, *P* < 0.005, Figure 3). However, composition of Cys, Phe, Gln, Lys, Leu, Asn, Trp, and Tyr increased significantly (*P* < 0.005, Figure 3).

### Heatmap Analysis of the Subsystem Categories

To explore the division between the species in terms of their metabolic capabilities and to highlight cold adaptations between the strains, heatmap analysis was employed to compare the subsystem groupings based on functional categories.

#### Genes Associated With Carbohydrates

**FIGURE 4:**
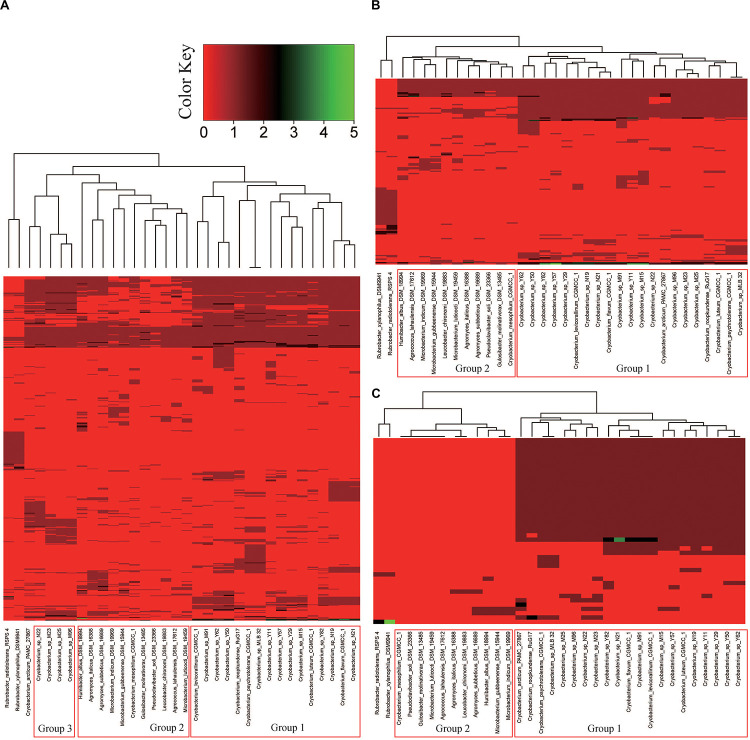
Hierarchical clustering analysis representing the presence/absence of gene families based on the RAST annotations. Genes in each panel: **(A)** “carbohydrates,” **(B)** “respiration,” and **(C)** “motility and chemotaxis.” The categories “carbohydrates,” “respiration,” and “motility and chemotaxis” contain 575, 154, and 43 genes, respectively. Details of the genes employed in each functional category are presented in Supplementary Table S6. Group 1 includes psychrophilic strains from poles and Tibetan Plateau, Group 2 includes *C. mesophilum* CGMCC 1.10440^T^ and the 10 genomes in the family Microbacteriaceae as described in Section “Materials and Methods,” and Group 3 includes five psychrophilic *Cryobacterium* strains.

Genes affiliated with the functional category “carbohydrates” of the Tibetan Plateau glacier isolates ranged from 559 (in strain Y50) to 377 (in strain Y29). The heatmap of RAST subsystem carbohydrates (genes involved in carbohydrate transport and metabolism) across all the *Cryobacterium* genomes resulted in a gene presence/absence matrix displaying three groups separated by the reference genomes (Supplementary Table S3 and Figure 4A). The cold adapted *Cryobacterium* strains in Group 1 and Group 3 form a single cluster separated from their mesophilic counterparts (Figure 4A). The difference in the proportion of carbohydrate utilization and metabolism between psychrophiles and mesophiles was demonstrated both inter genus and intra genus, as the only mesophilic strain *C. mesophilum* and the reference strains form a single separate cluster (Group 2 in Figure 4A). A similar clustering was also identified in the category “cofactors/vitamin/prosthetic groups/pigment” (Supplementary Figure S3).

#### Genes Associated With Respiration

Genes affiliated with the respiration category of the Tibetan Plateau glacier-derived genomes ranged from 45 (in strain N22) to 76 (in strains M19, Y50, and Y62). All the cold adapted *Cryobacterium* strains form a single cluster separate from both their inter and intra genus counterparts (Figure 4B). The mesophilic strain *C. mesophilum* and the reference strains also form a single separate cluster (Group 2) same as that in Figure 4A. This implies that the respiration profile of cold adapted *Cryobacterium* relatives has a strong consistency.

#### Genes Associated With Motility and Chemotaxis

Genes of the Tibetan Plateau glacier isolates that are involved in the category “motility and chemotaxis” ranged from 39 (in strain M91) to 49 (in strain Y82). Genes assigned to “motility and chemotaxis” are present in the cold adapted *Cryobacterium* strains but absent from reference strains (Figure 4C).

#### Specific Gene Families

**FIGURE 5:**
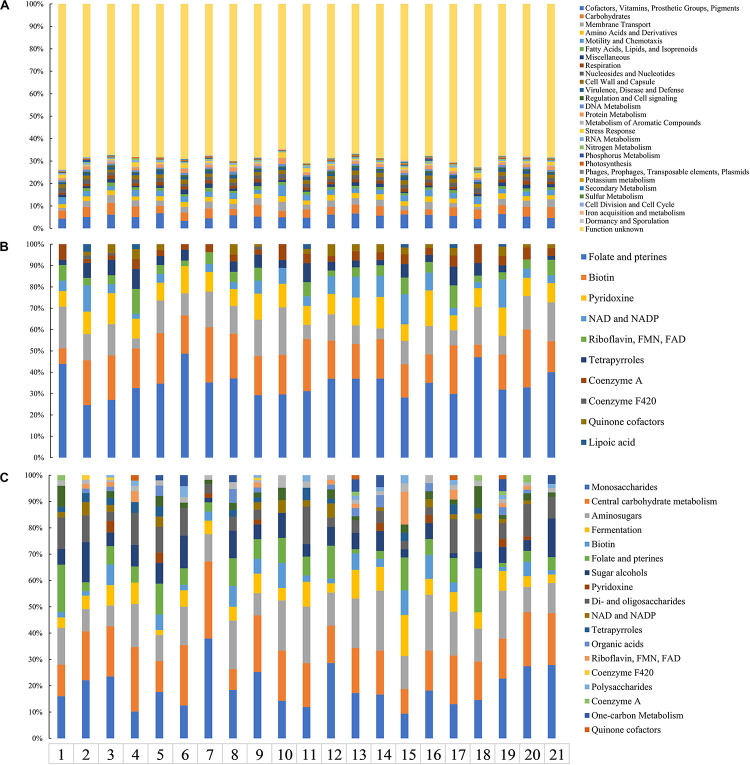
Distribution of specific genes harbored by psychrophilic strains 1, *C. arcticum*; 2, *Cryobacterium* sp. Y11; 3, *Cryobacterium* sp. Y50; 4, *Cryobacterium* sp. M96; 5, *Cryobacterium* sp. M15; 6, *Cryobacterium* sp. MLB-32; 7, *C. levicorallinum*; 8, *Cryobacterium* sp. Y29; 9, *Cryobacterium* sp. Y62; 10, *C. psychrotolerans*; 11, *Cryobacterium* sp. M25; 12, *Cryobacterium* sp. Y57; 13, *C. flavum*; 14, *Cryobacterium* sp. N19; 15, *C. luteum*; 16, *Cryobacterium* sp. Y82; 17, *Cryobacterium* sp. M23; 18, *Cryobacterium* sp. N22; 19, *Cryobacterium* sp. N21; 20, *Cryobacterium* sp. M91; 21, *C. roopkundense*. **(A)** Overall pattern of all genomes at the category level. Gene affiliation to subcategory level involved in **(B)** the category “cofactors, vitamins, prosthetic groups, pigments” and **(C)** the category “carbohydrates.”

Gene families specific to each strain (no paralogs in the FastOrtho analysis) within the genus *Cryobacterium* with respect to their mesophilic counterparts in family Microbacteriaceae ranged from 543 (in strain *C. psychrotolerans* CGMCC1.5382^T^) to 1567 (in strain Y50) (Supplementary Table S4). The proportion of function assignable genes to unknown function is approximately 1:2 (Figure 5A). This is not abnormal, since genes that have no hit in the RAST database are mostly classified as specific ones. The psychrophilic *Cryobacterium* species shared a similar affiliation of specific gene families to functional categories, which were mainly assigned to “cofactors, vitamins, prosthetic groups, pigments,” “carbohydrates,” and ABC transporters in “membrane transport.” Specific genes in the category “cofactors, vitamins, prosthetic groups, pigments” were predominated by the subcategory “folate, pterines, and biotin synthesis” (Figure 5B). While that in the category “carbohydrates” was most represented by the subcategory “monosaccharides metabolism” and “central carbohydrate metabolism” (Figure 5C).

### Cold Shock Genes

All the 14 genomes obtained from the present study and the references genomes have two predicted cold shock genes (Table 1). Neither numeric differences nor gene family differences (50% amino acid sequence similarity and 50% coverage) were observed between the psychrophilic *Cryobacterium* and the mesophilic *C. mesophilum*. We further compared the *Cryobacterium* species to reference strains at the family level. Surprisingly, no numeral advantage of cold adapted genes was detected in *Cryobacterium* genomes over the reference strains (Supplementary Table S3).

### Gene Gain and Loss

**FIGURE 6:**
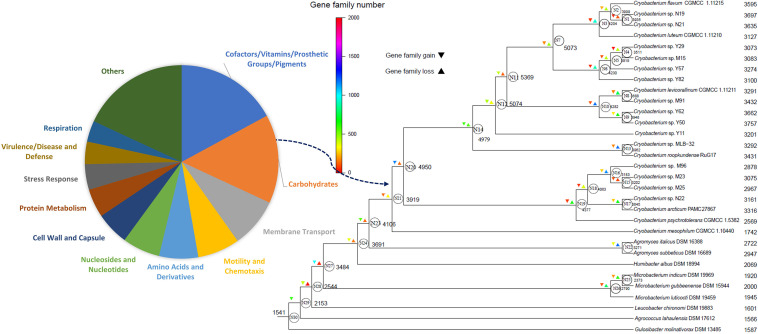
Gene gain and loss events during the evolution of *Cryobacterium* strains and the reference strains in the family Microbacteriaceae reconstructed using the COUNT software. The numbers next to each node and tip represent the genome size, and *N* represents node number. The log-scale color coding represents numbers of reconstructed gain and loss events of each lineage. Function distribution of the genes gained at node N20 is highlighted in the left panel.

The phylogenetic birth-and-death model imposed on the phylogenomic tree indicates a complicated evolutionary path of the *Cryobacterium* species with their relatives in the family Microbacteriaceae. The most recent common ancestor (∼1,541 genes) experienced early expansion in gene content, leading to the nodes N29, N28, N27, N24, N23, N23, N21, N20, N14, and N12 in Figure 6. After the early expansion, the extant lineages all experienced light gene loss (Figure 6). In comparison with the genomes of mesophilic Microbacteriaceae and *C. mesophilum*, the extant psychrophilic *Cryobacterium* species increased significantly in genome size (*P* < 0.01).

The remarkable surge in genome content was in the node N20, which gained 1,168 genes and lead to the psychrophilic *Cryobacterium* cluster. Genes gained at the node N20 with known function are involved in the categories of “cofactors, vitamins, prosthetic groups, pigments” (17%), “carbohydrates” (15%), “membrane transport” (8%), “motility and chemotaxis” (7%), and “amino acids and derivatives” (7%) (Figure 6).

Mesophilic strain *C. mesophilum* was located at the base of the *Cryobacterium* cluster with a streamlined genome (lost 2,190 genes, Supplementary Table S5). Despite a continuous increase in genome content of the nearest ancestor, *C. mesophilum* escaped the surge that occurred to its psychrophilic relatives, evolving directly toward genomes of 1,742 genes (Figure 6). The psychrophilic *Cryobacterium* ancestors (e.g., N14, N12, N11, and N7) show either genome surge or slightly expansion. The basal lineages diverged at node N22 show monotonically an increase in genome content since *Cryobacterium* ancestor.

**FIGURE 7:**
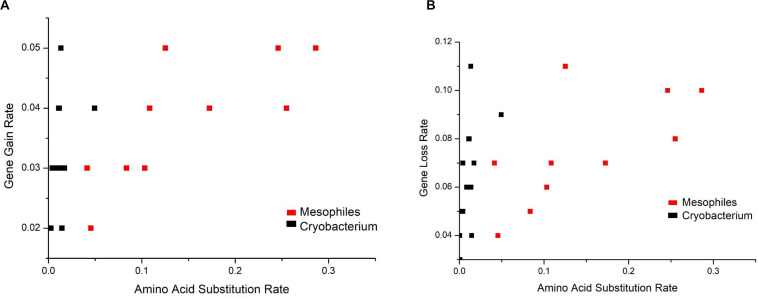
Analysis of gene gain rate **(A)** and gene loss rate **(B)** versus amino acid substitution rate. The phylogeny was constructed using RAxML. The amino acid substitution rate was extracted from the ML tree, and gene gain and loss rates were calculated in the ancestor reconstruction analysis.

Analysis of gene gain and gene loss rates versus amino acid substitution rates shows that gene gain and loss rates are at the same order of magnitude (Figures 7A,B). However, the amino acid substitution rates of *Cryobacterium* differed significantly (two orders of magnitude lower, *P* = 6.28E-8) from reference strains.

## Discussion

Study of microbial diversity in polar and high alpine regions has revealed a higher diversity of viruses, bacteria, and eukaryotic microbes than expected, and these extreme cold habitants are hot spots of microbial diversity and evolution (Anesio and Bellas, 2011; Margesin and Collins, 2019). In the present study, we compared 21 genomes of psychrophilic *Cryobacterium* to their mesophilic counterparts from the same family Microbacteriaceae with the aim to identify unique genomic features of the psychrophilic *Cryobacterium* strains isolated from polar and alpine environments. Psychrophilic *Cryobacterium* were revealed to have undergone more dynamic changes in genome content than their mesophilic counterparts, which likely led to the enhanced capabilities of resisting low temperatures, oligotrophy, and high UV radiation on glaciers.

### Universal Adaptation Strategies in Psychrophiles Are Selectively Adopted by *Cryobacterium*

Significant changes in amino acid composition were found in the genomes of psychrophilic *Cryobacterium*. Enzymes evolved specific compositional biases (i.e., changes in amino acid composition) to achieve structural flexibility that is required to afford enzyme activity at low temperatures (De Maayer et al., 2014). We identified a reduced use of acidic amino acids (aspartic acid and glutamic acid) and arginine, and an increased use of lysine in psychrophilic *Cryobacterium*, which is consistent with increased protein flexibility of psychrophiles at low temperatures (Ayala-del-Río et al., 2010; Siddiqui et al., 2013; De Maayer et al., 2014). However, we did not detect the reduced use of proline, the content of which generally decreased in other psychrophiles; this may be attributed to genus-specific adaptation (Rabus et al., 2004; Murray and Grzymski, 2007; De Maayer et al., 2014). Our results suggest that a combination of multi-taxa in comparative genomics is required to understand the universal adaptation strategy that can be identified on a large scale across psychrophiles (Raymond-Bouchard et al., 2018).

No apparent differences in the number and feature of cold shock genes were detected in psychrophilic *Cryobacterium*. Cold shock proteins (CSPs) are induced upon cold shock and are thought to bind a single-stranded RNA motif, resulting in reduced secondary structure formation in the RNA and thus increased translation efficiency (Rabus et al., 2004; De Maayer et al., 2014). It is important for bacteria to be able to synthesize proteins at low temperatures (Thomas and Cavicchioli, 2002; Rabus et al., 2004). Thus, cold shock genes are necessary for cold adaptation (Siddiqui et al., 2013). However, the number of CSP genes varied widely in the genomes of 22 bacterial and archaeal psychrophiles, and no quantitative superiority was observed in CSP genes harbored by psychrophiles (Siddiqui et al., 2013). Furthermore, proteomic profiles of psychrophile *Pseudoalteromonas haloplanktis* TAC 125 was reported to have no cold-induced proteins in response to a temperature shift from 18 to 4°C (Piette et al., 2012). The additional copies of CSP coding gene CspA in *Agromyces italicus* DSM 16388, *Gulosibacter molinativorax* DSM 13485, and *Humibacter albus* DSM 18994, in contrast to the psychrophilic *Cryobacterium* species, did not enable them to become psychrophiles (Manaia et al., 2004; Jurado et al., 2005; Vaz-Moreira et al., 2008). We also detected a decrease in the proportion of genes related to the category “respiration.” Low oxygen level was suggested as a trigger for enhancement of respiratory metabolism (Rintala et al., 2009). High altitude of Tibetan glacier connects with low oxygen content, but bacteria inhabiting there did not show an increase in the number of respiration genes. This might be because low temperature in snow and ice could increase the solubility of oxygen and result in less respiratory stress (Allen et al., 2009), which causes the decreasing of the proportion of genes in the category “respiratory’ in psychrophilic *Cryobacterium*. This speculation was supported by the fact that the gene proportion of the category “respiration” in Tibetan glacial strains (∼2.63%) are higher than that in polar regions (∼2.35%, *P* < 0.05). Our results, together with the evidence from these previous studies, indicate that gene multicopy may not be a key strategy used for cold adaptation.

### Niche Adaptation Governs the Genomic Contents in *Cryobacterium*

The enrichment of stressor response associated genes has been detected in glacier ice metagenome (Simon et al., 2009). Since the solubility of gasses increases rapidly at low temperatures, the ability to respond to reactive oxygen species is an essential function for organisms living at low temperatures (Simon et al., 2009). Not unexpectedly, one of the most striking differences in genome content between psychrophilic *Cryobacterium* strains and references is in the stress response genes. Genes related to “osmotic stress,” “oxidative stress,” and “periplasmic stress” are all involved in the category “stress response” (Overbeek et al., 2014).

The comparison of the subsystem groupings is based on functions, and differences can be detected from gene presence and absence matrixes, such as genes involved in “carbohydrates,” “respiration,” and “motility and chemotaxis.” However, the psychrophilic *Cryobacterium* genomes are very divergent, with more than 39 genes involved in “motility and chemotaxis” while the mesophilic counterparts have less than 10 genes of the same category. Literature has rarely reported the correlation between the presence of genes involved in “motility and chemotaxis” and the ability of cold adaptation (De Maayer et al., 2014). For the psychrophilic *Cryobacterium* strains, the presence of genes involved in motility and chemotaxis may be one of the key genomic advantages for coping with cold environments. This may be especially true for the isolates from ice cores (M/N/Y series of strains in this study), as genes involved in “motility and chemotaxis” can help them to move toward interstitial veins that exist at the grain boundaries of ice crystals and thus acquire metabolites (Price, 2000; Amato and Christner, 2009; Mackelprang et al., 2017).

Niche adaptation appears to play a significant role in governing the genetic content of the *Cryobacterium* strains isolated from Tibetan Plateau, Antarctic, and Arctic as indicated by the distribution pattern of specific gene families. Studies performed on *Lactococcus* and *Novosphingobium* genomes also suggest that there are habitat-specific genes (Kelleher et al., 2017; Kumar et al., 2017), for example, strengthening in ultraviolet radiation resistance and preferential utilization of simpler forms of carbohydrates (Singh et al., 2014; Shen et al., 2019). Niche adaptation relies heavily on the acquisition of new metabolic capabilities as well as the loss of unnecessary functions (Feng et al., 2014). Comparison of whole genomes of psychrophilic *Cryobacterium* strains with respect to the reference strains in this study revealed the presence of between 543 and 1,422 specific gene families for *Cryobacterium* strains. Of these specific gene families, all the genomes have similar dominating functional groups (“cofactors, vitamins, prosthetic groups, pigments,” “carbohydrates,” “membrane transport,” “motility and chemotaxis,” and “amino acids and derivatives”), reflecting the resultant adaptive changes in the genomes of *Cryobacterium* strains in response to harsh glacial environments. The distribution of specific genes also differed between psychrophilic *Cryobacterium* strains and the reference strains. For example, the most dominant functional group of *C. mesophilum* is “amino acids and derivatives” (Supplementary Figure S2). Our results revealed a different distribution of functional genes between psychrophilic *Cryobacterium* strains and the reference strains, and the biased distribution of functional genes may be due to the high genome plasticity of the cold adapted microorganisms (Allen et al., 2009).

### Psychrophilic *Cryobacterium* Evolved by Gene Gains and Losses

We used amino acid substitution rate as a proxy of genome evolution at the basal level, i.e., accumulation of natural neutral mutations that occur across all *Cryobacterium* genomes at an equal rate. This is a passive process leading to genome variations without affecting fitness. In contrast, gene gain and loss are more proactive processes leading to rapid niche differentiation. The large difference in the rates of these two different evolutionary processes highlights the importance of gene gain/loss events during the evolution of psychrophilic *Cryobacterium*.

Based on the genome dynamics analysis, we identified a genomic expansion event in psychrophilic *Cryobacterium* genomes during the initial period, which may have facilitated the species in adapting to cold environments, like Tibetan Plateau glaciers, Himalayan lake, and Antarctica sedimentary rock, which were further divided into subgroups. The early surge in gene content, leading to the N20 node, may eventually lead to the origin of psychrophilic *Cryobacterium* species. This was supported by the fact that the only mesophilic strain *C. mesophilum* CGMCC1.10440^T^ was not involved in this surge.

Genes gained at the nod N20 are predominated by the functional category “cofactors, vitamins, prosthetic groups, pigments.” Genes involved in this category process a series of key compounds (e.g., folate, pterines, biotin, pyridoxine, riboflavin, and carotenoids) in bacteria coping with environmental stressors. The vast surge in the genome content of the common ancestor of psychrophilic *Cryobacterium* may be induced by the environmental pressure. Adaptation to environmental stressor is dependent on the time necessary to accumulate mutations or new genes and the degree of perturbation (Jansson and Hofmockel, 2020). Our study revealed that microorganisms in low-temperature environments with long generation times (Mackelprang et al., 2017) gaining genes via horizontal transfer may be the most effective way. The added genome content can help psychrophilic *Cryobacterium* to cope with low temperatures, high ultraviolet radiation, and oligotrophic glacial environments (Domingues and Nielsen, 2017). This is consistent with phenotypic characteristics commonly observed in microbes from cold habitats, for example, the pigmentation of microorganisms isolated from the Northern Schneeferner, Tibetan Plateau, and Greenland (Miteva et al., 2004; Simon et al., 2009; Shen et al., 2014). The classification of specific genes and genes gained at the nod N20 is similar, which may suggest that *Cryobacterium*-specific genes are likely the genes that provided *Cryobacterium* with psychrophilic functionality.

Genes gained by psychrophilic *Cryobacterium* spp. are mostly involved in “cofactors, vitamins, prosthetic groups, pigments,” while genes gained by the psychrophilic bacterium *Psychroflexus torquis* ATCC 700755 from sea ice are mostly referred to exopolysaccharide (EPS) and polyunsaturated fatty acid (PUFA) biosynthesis when compared to its closely related mesophilic sister species (Feng et al., 2014). This inconsistency of functional genes gained by cold adapted species indicates different key environmental factors existing for shaping the genome content of bacteria from alpine glacier and sea ice. The intensive ultraviolet radiation may be an underestimated environmental factor in addition to low temperatures, high osmotic pressures, and low nutrient availability in glaciers on the Tibetan Plateau, which together exert a constant and strong evolutionary pressure on the genomes of glacial bacteria. The traits of anti-ultraviolet radiation were also illustrated in mammals living on Tibetan Plateau (Huerta-Sanchez et al., 2014; Yang et al., 2017). For example, the folate-increasing allele of rs1801133 has an increased frequency in Tibetans, which is probably a consequence of adaptation to high UV radiation (Yang et al., 2017). The folate genes are also involved in the category “cofactors, vitamins, prosthetic groups, pigments” in bacterial genomes (Overbeek et al., 2014), the main category gained by psychrophilic *Cryobacterium* genomes. Our results support the view that low-temperature habitats are hot spots of microbial evolution (Anesio and Bellas, 2011).

## Conclusion

Our study suggests that cold adaptation appears to play a significant part in governing the genome content of glacial bacteria. This is supported by the findings that the vast surge and biased gene gains by the common ancestor of *Cryobacterium* occurred after the divergence of the mesophilic *C. mesophilum*. Considering the surge in genome content and the low amino acid substitution rate, the transfer of genes between glacial bacteria (e.g., mediated by viruses, plasmids, and other mobile elements) may be higher than previously thought despite their long generation time. Further studies on the gene expression patterns in both psychrophilic and mesophilic *Cryobacterium* are required to establish the direct link between genome differences and cold adaptation.

## Data Availability Statement

The datasets generated for this study can be found in the DDBJ/ENA/GenBank PJJJ00000000–PJJX00000000.

## Author Contributions

YL designed the study. LS performed the bioinformatics analysis. TX performed the lab experiments. BX and NW collected the samples. YL, LS, and YZ together wrote the manuscript. All authors approved the final version.

## Conflict of Interest

The authors declare that the research was conducted in the absence of any commercial or financial relationships that could be construed as a potential conflict of interest.

## Footnotes

1 http://enews.patricbrc.org/fastortho/
