# Supplementary material for: Genomic Insights of Cryobacterium Isolated From Ice Core Reveal Genome Dynamics for Adaptation in Glacier
Source: Front Microbiol. 2020 Jul 14;11:1530. doi: 10.3389/fmicb.2020.01530 (PMC7381226; doi:10.3389/fmicb.2020.01530)
Supplement: Supplementary file 1 [file Data_Sheet_1.docx]

**Genomic insights of *Cryobacterium* isolated from ice core reveal genome dynamics for adaptation in glacier**

Yongqin Liu^1, 2, 6*^, Liang Shen^1, 5^, Yonghui Zeng^3^, Tingting Xing^1, 6^, Baiqing Xu^1, 2^, Ninglian Wang^2, 4^

^1^Key Laboratory of Tibetan Environment Changes and Land Surface Processes, Institute of Tibetan Plateau Research, Chinese Academy of Sciences, Beijing, 100085, China

^2^CAS Center for Excellence in Tibetan Plateau Earth Sciences, Beijing, 100085, China

^3^Department of Environmental Science, Aarhus University, Roskilde 4000, Denmark

^4^College of Urban and Environmental Science, Northwest University, Xian, 710069, China

^5^College of Life Sciences, Anhui Normal University, Wuhu, 241000, China

^6^University of Chinese Academy of Sciences, Beijing 100101, China

*Author for correspondence:

Yongqin Liu, yqliu@itpcas.ac.cn, Institute of Tibetan Plateau Research, Chinese Academy of Sciences, Beijing, 100101, China

Fig. S1 Phylogenetic clustering of 32 Microbacteriaceae strains. Phylogeny is based on 16S rRNA gene sequences with 1,000 bootstraps using MEGA10. *Rubrobacter xylanophilus* DSM9941 and *R. radiotolerans* RSPS-4 were used as out-groups. Bar 0.02 accumulated changes per nucleotide.

Fig. S2 Distribution of specific genes harbored by reference strains.


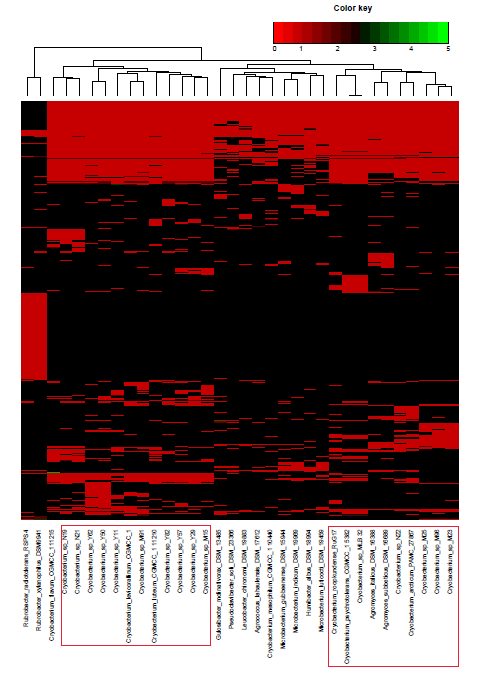


Fig. S3 Hierarchical clustering analysis representing the presence/absence of gene families from the category ‘cofactors/vitamin/prosthetic groups/pigmen’ based on the RAST annotations. Psychrophilic *Cryobacterium* were in red frame.
